# EL_PSSM-RT: DNA-binding residue prediction by integrating ensemble learning with PSSM Relation Transformation

**DOI:** 10.1186/s12859-017-1792-8

**Published:** 2017-08-29

**Authors:** Jiyun Zhou, Qin Lu, Ruifeng Xu, Yulan He, Hongpeng Wang

**Affiliations:** 1 School of Computer Science and Technology, Harbin Institute of Technology Shenzhen Graduate School, HIT Campus Shenzhen University Town, Xili, Shenzhen, Guangdong 518055 China; 2 Department of Computing, the Hong Kong Polytechnic University, Kowloon, Hong Kong; 3 Shenzhen Engineering Laboratory of Performance Robots at Digital Stage, Shenzhen Graduate School, Harbin Institute of Technology, Shenzhen, China; 4 School of Engineering and Applied Science, Aston University, Birmingham, UK

**Keywords:** DNA-protein interaction, DNA-binding residue, PSSM, Ensemble learning, SVM, Random forest, Relation transformation

## Abstract

**Background:**

Prediction of DNA-binding residue is important for understanding the protein-DNA recognition mechanism. Many computational methods have been proposed for the prediction, but most of them do not consider the relationships of evolutionary information between residues.

**Results:**

In this paper, we first propose a novel residue encoding method, referred to as the Position Specific Score Matrix (PSSM) Relation Transformation (PSSM-RT), to encode residues by utilizing the relationships of evolutionary information between residues. PDNA-62 and PDNA-224 are used to evaluate PSSM-RT and two existing PSSM encoding methods by five-fold cross-validation. Performance evaluations indicate that PSSM-RT is more effective than previous methods. This validates the point that the relationship of evolutionary information between residues is indeed useful in DNA-binding residue prediction. An ensemble learning classifier (EL_PSSM-RT) is also proposed by combining ensemble learning model and PSSM-RT to better handle the imbalance between binding and non-binding residues in datasets. EL_PSSM-RT is evaluated by five-fold cross-validation using PDNA-62 and PDNA-224 as well as two independent datasets TS-72 and TS-61. Performance comparisons with existing predictors on the four datasets demonstrate that EL_PSSM-RT is the best-performing method among all the predicting methods with improvement between 0.02–0.07 for MCC, 4.18–21.47% for ST and 0.013–0.131 for AUC. Furthermore, we analyze the importance of the pair-relationships extracted by PSSM-RT and the results validates the usefulness of PSSM-RT for encoding DNA-binding residues.

**Conclusions:**

We propose a novel prediction method for the prediction of DNA-binding residue with the inclusion of relationship of evolutionary information and ensemble learning. Performance evaluation shows that the relationship of evolutionary information between residues is indeed useful in DNA-binding residue prediction and ensemble learning can be used to address the data imbalance issue between binding and non-binding residues. A web service of EL_PSSM-RT (http://hlt.hitsz.edu.cn:8080/PSSM-RT_SVM/) is provided for free access to the biological research community.

**Electronic supplementary material:**

The online version of this article (doi:10.1186/s12859-017-1792-8) contains supplementary material, which is available to authorized users.

## Background

DNA-binding proteins play an important role in many essential biological processes such as DNA replication, recombination, repair, gene transcription and expression [1]. It has been reported that 2–3% of a prokaryotic genome and 6–7% of a eukaryotic genome encode DNA-binding proteins [2, 3]. As the interactions between proteins and DNAs are mainly formed by the immediate contacts [4], the identification of residues involved in the contacts is important for understanding the mechanism between them. Moreover, the identification of DNA-binding residues can also contribute to the understanding of the pathogenesis of diseases. Bullock and Fersht [5] have reported that mutations of some DNA-binding residues on proteins may be associated with some diseases. For example, the mutations of DNA-binding residues on the tumor repressor protein P53 may predispose individuals to cancer [5]. Many experimental techniques have been proposed to distinguish DNA-binding residues including electrophoretic mobility shift assays (EMSAs) [6, 7], nuclear magnetic resonance (NMR) spectroscopy [8], X-ray crystallography [9], peptide nucleic acid (PNA)-assisted identification of RNA binding proteins (RBPs) (PAIR) [10], MicroChIP [11], Fast ChIP [12], and conventional chromatin immunoprecipitation (ChIP) [13]. However, the experimental methods are very expensive and time-consuming. With the rapid accumulation of protein sequences, there is an urgent need to develop computational methods for the identification of DNA-binding residues.

For DNA-binding residue prediction, many computational methods have been proposed in recent years. The features used in these prediction methods include three types: sequence features, structure features and evolutionary features. In the early stage, the evolutionary features are not easy to get due to the limitation of computing power, so the predictors were developed mainly based on either structure information or sequence features, or a combination of them. For instance, the Support Vector Machine (SVM) classifier developed by Ahmad et al. [14] utilized only sequence features, such as the local amino acid composition and solvent accessible surface area. The classifier built by Tsuchiya et al. [15] used only structure features, such as electrostatic potential on the surface and the shape of the molecular surface. The DNA-binding residue classifier proposed by Bhardwaj [16] et al. used both sequence and structure information, such as solvent accessibility, local composition, net charge, and electrostatic potentials. The later SVM classifier proposed by Bhardwaj et al. [17] used structure features such as the net charge of a residue, occurrence in a cationic patch, and the average potential on a residue in addition to the features used in their previous work [16]. The major limitation for the methods described above is that they did not use any evolutionary information which has been reported to be helpful for protein function prediction [18–20]. Thus, incorporating evolutionary information into the identification of DNA-binding residues may potentially improve its identification accuracy.

With the improvement of computing power, the use of evolutionary features becomes easier. Thus, more methods are now using evolutionary features for the prediction. Position Specific Score Matrix (PSSM) is a common representation of the evolutionary features and is used in the prediction methods in two ways: (1) combination methods that encode residues by combining evolutionary information and physiochemical properties and (2) concatenation methods that encode residues by concatenating the PSSM (Position Specific Score Matrix) scores in the sliding window. In combination methods, PSSM is combined with physiochemical properties to calculate the feature values for every residue. For instance, the encoding method proposed by Wang et al. [21] combined the BLAST-based conservation scores generated by sequence alignment and several biochemical properties to calculate the feature values for residues. The later encoding method proposed by his group [19] combined three physicochemical features including hydrophobicity, side chain pKa value and molecular mass and frequency profile to calculate the physicochemical feature values for the target residue and its context residues. The mean and the standard deviation of the three physicochemical features are used to construct the feature space. The encoding method proposed by Ma et al. [22] combined PSSM and four physicochemical properties including the lone electron pairs, hydrophobicity, side chain pKa value and molecular mass.

Concatenation methods usually concatenate the PSSM scores of all the residues in the sliding window to encode residues. For instance, Ahmad and Sarai’s work [20] concatenated all the PSSM scores of residues within the sliding window of the target residue to construct the feature vector. Then the concatenation method proposed by Ahmad and Sarai [20] were used by many classifiers. For example, the SVM classifier proposed by Kuznetsov et al. [23] was developed by combining the concatenation method, sequence features and structure features. The predictor, called SVM-PSSM, proposed by Ho et al. [24] was developed by the concatenation method. The SVM classifier proposed by Ofran et al. [1] was developed by integrating the concatenation method and sequence features including predicted solvent accessibility, and predicted secondary structure.

It should be noted that both current combination methods and concatenation methods did not include the relationships of evolutionary information between residues. However, many works on protein function and structure prediction have already shown that the relationships of evolutionary information between residues are important [25, 26], we propose a method to include the relationship of evolutionary information as features for the prediction of DNA-binding residue. The novel encoding method, referred to as the PSSM Relationship Transformation (PSSM-RT), encodes residues by incorporating the relationships of evolutionary information between residues. In addition to evolutionary information, sequence features, physicochemical features and structure features are also important for the prediction. However, as the structure features for most of the proteins are unavailable, we do not include structure feature in this work. In this paper, we include PSSM-RT, sequence features and physicochemical features to encode residues. Additionally, for DNA-binding residue prediction, there are much more non-binding residues than binding residues in protein sequences. However, most of the previous methods cannot take advantages of the abundant number of non-binding residues for the prediction. In this work, we propose an ensemble learning model by combining SVM and Random Forest to make good use of the abundant number of non-binding residues. By combining PSSM-RT, sequence features and physicochemical features with the ensemble learning model, we develop a new classifier for DNA-binding residue prediction, referred to as EL_PSSM-RT. A web service of EL_PSSM-RT (http://hlt.hitsz.edu.cn:8080/PSSM-RT_SVM/) is made available for free access by the biological research community.

## Methods

As shown by many recently published works [27–30], a complete prediction model in bioinformatics should contain the following five components: validation benchmark dataset(s), an effective feature extraction procedure, an efficient predicting algorithm, a set of fair evaluation criteria and a web service to make the developed predictor publicly accessible. In the following text, we will describe the five components of our proposed EL_PSSM-RT in details.

### Datasets

In order to evaluate the prediction performance of EL_PSSM-RT for DNA-binding residue prediction and to compare it with other existing state-of-the-art prediction classifiers, we use two benchmarking datasets and two independent datasets.

The first benchmarking dataset, PDNA-62, was constructed by Ahmad et al. [14] and contains 67 proteins from the Protein Data Bank (PDB) [31]. The similarity between any two proteins in PDNA-62 is less than 25%. The second benchmarking dataset, PDNA-224, is a recently developed dataset for DNA-binding residue prediction [32], which contains 224 protein sequences. The 224 protein sequences are extracted from 224 protein-DNA complexes retrieved from PDB [31] by using the cut-off pair-wise sequence similarity of 25%. The evaluations on these two benchmarking datasets are conducted by five-fold cross-validation. To compare with other methods that were not evaluated on the above two datasets, two independent test datasets are used to evaluate the prediction accuracy of EL_PSSM-RT. The first independent dataset, TS-72, contains 72 protein chains from 60 protein-DNA complexes which were selected from the DBP-337 dataset. DBP-337 was recently proposed by Ma et al. [33] and contains 337 proteins from PDB [31]. The sequence identity between any two chains in DBP-337 is less than 25%. The remaining 265 protein chains in DBP-337, referred to as TR265, are used as the training dataset for the testing on TS-72. The second independent dataset, TS-61, is a novel independent dataset with 61 sequences constructed in this paper by applying a two-step procedure: (1) retrieving protein-DNA complexes from PDB [31]; (2) screening the sequences with cut-off pair-wise sequence similarity of 25% and removing the sequences having > 25% sequence similarity with the sequences in PDNA-62, PDNA-224 and TS-72 using CD-HIT [34]. CD-HIT is a local alignment method and short word filter [35, 36] is used to cluster sequences. In CD-HIT, the clustering sequence identity threshold and word length are set as 0.25 and 2, respectively. By using the short word requirement, CD-HIT skips most pairwise alignments because it knows that the similarity of two sequences is below certain threshold by simple word counting. For the testing on TS-61, PDNA-62 is used as the training dataset. The PDB id and the chain id of the protein sequences in these four datasets are listed in the part A, B, C, D of the Additional file 1, respectively.

In the above 4 datasets, positive and negative samples are defined by the following criterion [18, 37, 38]: a residue in a protein is regarded as a binding residue if the side chain or the backbone atoms of the residue falls within a cutoff distance of 3.5 Å from any atom of the partner DNA molecule in the complex; Otherwise, the residue is considered a non-binding residue. The number of positive samples and negative samples of the four datasets are shown in Table 1.

**Table 1:** Number of the positive samples and negative samples of the four datasets

Dataset	PDNA-62	PDNA-224	TS-72	TS-61
Binding residue	1215	3778	1040	1078
Non-binding residue	6948	53,570	13,226	13,175

### Evaluation metrics

In order to evaluate the performance of EL_PSSM-RT for DNA-binding residue prediction, Sensitivity (SN), Specificity (SP), Strength (ST), Accuracy (ACC), and Mathews Correlation Coefficient (MCC) are used as performance metrics. They are typical evaluation metrics in bioinformatics and have been widely used by many works. The five metrics can be calculated according to the following formula

1 $$ \mathrm{SN}= TP/\left( TP+ FN\right) $$

2 $$ \mathrm{SP}= TN/\left( TN+ FP\right) $$

3 $$ \mathrm{ST}=\left(\mathrm{SN}+\mathrm{SP}\right)/2 $$

4 $$ \mathrm{ACC}=\left( TP+ TN\right)/\left( TP+ FP+ TN+ FN\right) $$

5 $$ \mathrm{MCC}=\left({TP}^{\ast } TN-{FP}^{\ast } FN\right)/\sqrt{{\left( TP+ FN\right)}^{\ast }{\left( TP+ FP\right)}^{\ast }{\left( TN+ FP\right)}^{\ast}\left( TN+ FN\right)} $$

where *TP* is the number of true positives, *TN* is the number of true negatives, *FP* is the number of false positives, and *FN* is the number of false negatives.

Since all the four datasets have much more negative training examples than positive training examples, using ACC alone may produce biased results, for example simply classifying all the test samples as non-binding residues will give a very high ACC value. Many literatures have indicated that ST, the average of SN and SP, can give a more appropriate evaluation for a classifier when the numbers of positive and negative samples are unbalanced [14, 38, 39]. Additionally, since MCC can measure the matching degree between the predicted results and the real results, it is also an appropriate evaluation metric. Moreover, the Receiver Operating Characteristic (ROC) curve [40] and the area under ROC curve (AUC) [41] are two more commonly used metrics for performance evaluation on imbalanced data sets. The ROC curve is drawn by plotting the true positive rates (i.e. sensitivity) against the false positive rates (i.e. 1-specificity) calculated by changing the classification threshold for predictors. AUC is the area under the ROC curve with values limited to the closed interval between −1.0 and 1.0. An AUC of 1.0 and 0.5 indicate the best performance and a random performance, respectively. Therefore, ST, MCC, AUC and ROC are used as the main performance measures and the other three metrics are used for references only.

### Sequence context

In DNA-binding residue prediction, residues are the samples for training and testing [16, 42]. Apart from a target residue, its adjacent residues also have a significant impact on its function. So, the sequence context of the target residue needs to be considered in the prediction. In order to use the sequence context for prediction, we define residue-wise data instances by a sliding window with size *w.* The sliding window is a sequence fragment with the target residue positioned in the middle and (*w*-1)/2 neighboring residues on either side. All the residues in the sliding window except the target residue are considered as the sequence context. The (*w*-1)/2 neighboring residues on the left side and the right side are referred to as the left sequence context and the right sequence context, respectively. The length of the sliding window, *w*, should be an odd number to be set experimentally.

Given a protein sequence P of length *L* denoted as

6 $$ \mathrm{P}={\mathrm{R}}_1{\mathrm{R}}_2{\mathrm{R}}_3{\mathrm{R}}_4{\mathrm{R}}_5{\mathrm{R}}_6\cdots {\mathrm{R}}_{i-1}{\mathrm{R}}_i{\mathrm{R}}_{i+1}\cdots {\mathrm{R}}_L, $$

where R_1_ represents the first residue of the protein sequence P, R_2_ represents the second residue and so forth. The residue-wise instance F_*i*_ for target residue R_*i*_ can be represented as

7 $$ {\mathrm{F}}_i={\mathrm{R}}_{i-\frac{w-1}{2}}{\mathrm{R}}_{i-\frac{w-3}{2}}\cdots {\mathrm{R}}_{i-1}{\mathrm{R}}_i{\mathrm{R}}_{i+1}\cdots {\mathrm{R}}_{i+\frac{w-3}{2}}{\mathrm{R}}_{i+\frac{w-1}{2}}, $$

where all the residues in the residue-wise instance F_*i*_ except the target residue R_*i*_ define its sequence context.

### Features of data instance

Evolutionary information is produced by the evolutionary processes and it is important for protein structure and function prediction. PSSM is a common representation for evolutionary information and has been used in many bioinformatics studies including protein functionality annotation and protein structure prediction [43–47]. For every protein sequence in this study, its PSSM is calculated from multiple sequence alignments produced by running the PSI-BLAST program [48] to search the non-redundant (NR) database through three iterations with the E-value cutoff at 0.001. For a protein with length *L*, PSSM is usually represented as a matrix with *L* × 20 dimensions. 20 denote the 20 standard types of residues. For the sequence fragment F_*i*_ using representation defined in Formula (7), its PSSM can be represented as a matrix with dimensions *w* × 20. Thus, the PSSM of the residue-wise instance F_*i*_ for the target residue R_*i*_ can be formulated as

8 $$ \mathrm{PSS}{\mathbf{M}}_{{\mathrm{F}}_i}=\left[\begin{array}{ccccc}\hfill {S}_{i-\frac{w-1}{2},1}\hfill & \hfill \cdots \hfill & \hfill {S}_{i-\frac{w-1}{2},r}\hfill & \hfill \cdots \hfill & \hfill {S}_{i-\frac{w-1}{2},20}\hfill \\ {}\hfill \vdots \hfill & \hfill \cdots \hfill & \hfill \vdots \hfill & \hfill \cdots \hfill & \hfill \vdots \hfill \\ {}\hfill {S}_{i,1}\hfill & \hfill \cdots \hfill & \hfill {S}_{i,r}\hfill & \hfill \cdots \hfill & \hfill {S}_{i,20}\hfill \\ {}\hfill \vdots \hfill & \hfill \cdots \hfill & \hfill \vdots \hfill & \hfill \cdots \hfill & \hfill \vdots \hfill \\ {}\hfill {S}_{i+\frac{w-1}{2},1}\hfill & \hfill \cdots \hfill & \hfill {S}_{i+\frac{w-1}{2},r}\hfill & \hfill \cdots \hfill & \hfill {S}_{i+\frac{w-1}{2},20}\hfill \end{array}\right], $$

where *S*_*i,r*_ is the conservative score of residue type *r* at position *i* in the sequence fragment.

Before PSSM-RT is calculated, the conservative scores in PSSM should be normalized between 0 and 1. Thus, for a given *S*_*i,r*_, its normalized value *S*_*i,r*_^(N)^ can be expressed by a logistic function given below

9 $$ {S}_{i,r}^{\left(\mathrm{N}\right)}=\frac{1}{1+{e}^{-{S}_{i,r}}}, $$

PSSM-RT contains three categories of features: residue conservations, pair-relationships and multi-relationships. The residue conservations contain the PSSM scores of the target residue and its context residues. The pair-relationship is defined as the relationship of evolutionary information between two positions, for example, the pair-relationship between the residue *r*_1_ of position *i* and the residue *r*_2_ of position *j* is calculated as

10 $$ \mathrm{PSSM}\hbox{-} \mathrm{RT}\left(i,j,{r}_1,{r}_2\right)={S_{i,{r}_2}^{\left(\mathrm{N}\right)}}^{\ast }{S}_{j,{r}_1}^{\left(\mathrm{N}\right)}, $$

As every position in a residue-wise data instance has conservative scores for the 20 standard residue type, 400 types of relationships can be calculated for any two positions.

As the target position in a residue-wise data instance is influenced by all its context positions, the all pair-relationships between the target position and its context positions needs to be included in the prediction. Thus the pair-relationship for a residue-wise data instance is defined as the sum of pair-relationship between the target position and all its context positions. For example, the pair-relationship between residue *r*_1_ and residue *r*_2_ for a residue-wise data instance with *i* as its target position is formulated as

11 $$ \mathrm{PSSM}\hbox{-} \mathrm{RT}\left(i,{r}_1,{r}_2\right)=\sum_j\mathrm{PSSM}\hbox{-} \mathrm{RT}\left(i,j,{r}_1,{r}_2\right), $$

where *j* is the context position of the target position.

Multi-relationships are the evolutionary information relationships between multiple residues. We consider two kinds of multi-relationships: the left multi-relationships that include the relationships between the target residue and its left context residues and the right multi-relationships that include the relationships between the target residue and its right context residues. For residue *r*, the left multi-relationship of residue-wise data instance at target position *i* is formulated as

12 $$ \mathrm{PSSM}\hbox{-} \mathrm{RT}\left(i,r\right)_{left}=\sum_{k=i-\frac{w-1}{2}}^i{S}_{k,r}^{\left(\mathrm{N}\right)}. $$

For residue *r*, the right multi-relationship of residue-wise data instance at target position *i* is formulated as

13 $$ \mathrm{PSSM}\hbox{-} \mathrm{RT}\left(i,r\right)_{right}=\sum_{k=i}^{i+\frac{w-1}{2}}{S}_{k,r}^{\left(\mathrm{N}\right)}. $$

Thus, the dimension of the feature space constructed by PSSM-RT is (20*w + 20*20 + 2*20).

In addition to PSSM-RT, there are two other types of features that are used in this work: sequence features and physiochemical features. Sequence features given in the datasets include amino acid composition, predicted secondary structure, predicted solvent accessible area, and identity of the target residue. Physiochemical features include pKa values of amino group, pKa values of carboxyl group, electron-ion interaction potential (EIIP) [49], number of lone electron pairs(LEPs), Wiener index [50], molecular mass [50], side chain pKa value, and hydrophobicity index. The predicted secondary structure and predicted solvent accessible area are obtained by applying PSIPRED [51] and SABLE [52–54], respectively.

### Ensemble learning

Ensemble learning is now an active area of research in machine learning and pattern recognition. Ensemble learning first learns several base predictors from the training dataset and then combines them into an ensemble predictor. Ensemble learning aims to take advantage of the different learning ability of the different base predictors. There are three widely used ensemble strategies to train base predictors: training by different data subsets, training from different feature subsets and training by different classification algorithms.

**Fig. 1:**
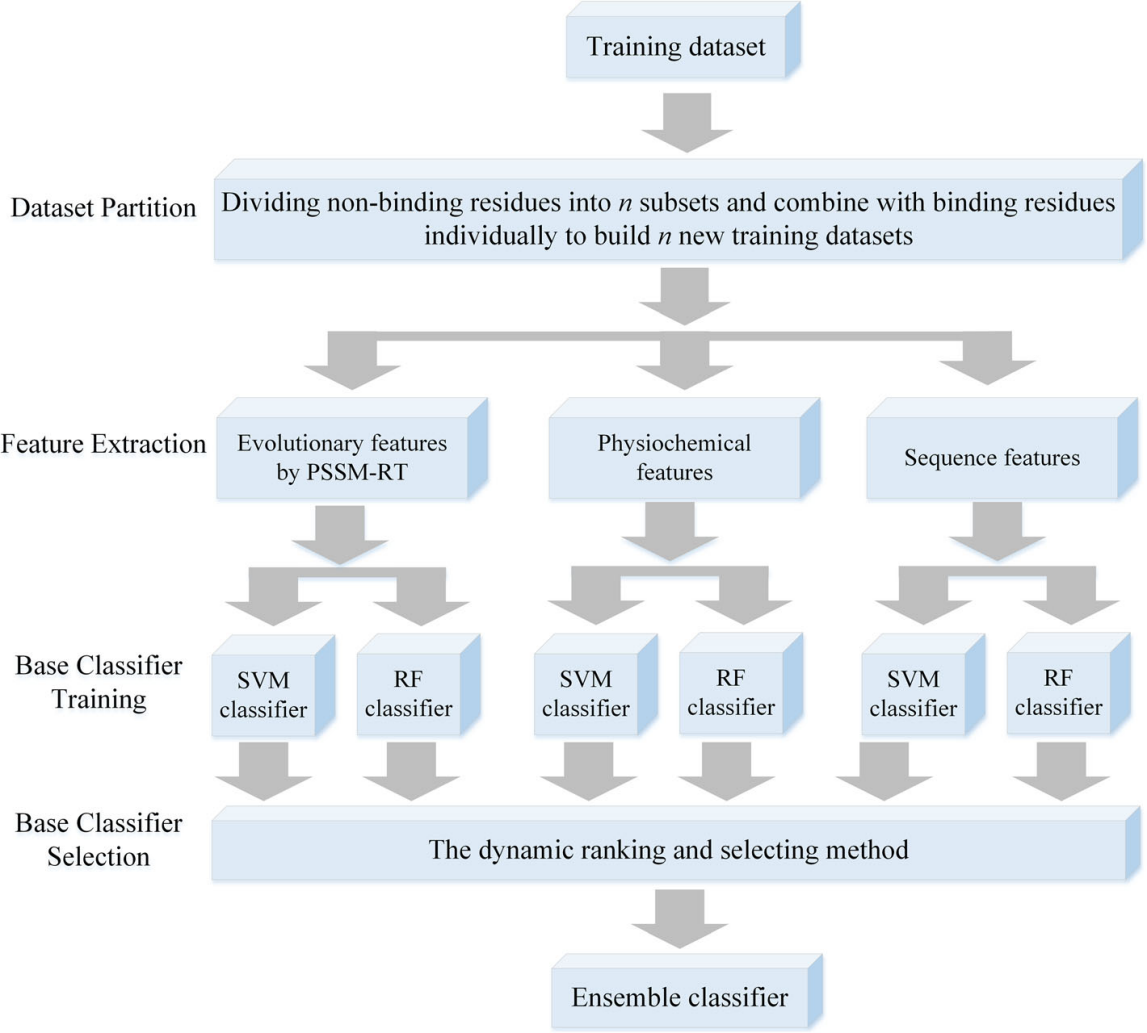
The framework diagram of EL_PSSM-RT. EL_PSSM-RT contains 4 steps. The first step is to divide the non-binding residues in the training dataset into *n* subsets and to construct *n* new training datasets by combining the *n* subsets of non-binding residues and binding residues individually. The secondary step is to extract the three categories of features for all the residues. The third step is to train both SVM classifier and Random Forest classifier by each category of features on every training subset. The fourth step is to use a dynamic ranking and selecting method to select the based predictors with the largest diversity between each other to build the ensemble predictor

In DNA-binding residue prediction, non-binding residues outnumber binding residues by a large margin. In order to get a balanced dataset for training, many predictors chose to discard a large part of non-binding residues [33]. However, discarded non-binding residues may potentially be useful information to improve prediction performance. In order to better use all the data available, we propose to use ensemble learning by combining all the three ensemble strategies. And then use our proposed method, referred to as EL_PSSM-RT, to combine the ensemble learning model with PSSM-RT. The system architecture of EL_PSSM-RT is shown in Fig. 1. Note that EL_PSSM-RT contains 4 steps: Dataset Partition, Feature Extraction, Base Classifier Training and Base Classifier Selection. In Step 1 of Dataset Partition, the non-binding residues in the training dataset are first partitioned into *n* non-overlapping subsets with the number of samples approximately equal to that of all the binding residues. Then, *n* new balanced training datasets are formed by adding the binding residues into the *n* subsets non-binding residues. In Step 2 of Feature Extraction, three categories of features are extracted for residues including sequence features, physiochemical features, and evolutionary information extracted by PSSM-RT. In Step 3 of Base Classifier Training, both the SVM classifier and the Random Forest classifier are used by each category of features on every newly formed training dataset. SVM and Random Forest are used because they are proven to have good predicting performances for DNA-binding residue prediction [18, 19, 55]. Thus, 6*n (2*3*n) base predictors are trained in this step. In Step 4 of Base Classifier Selection, a diversity based dynamic ranking and selecting method is designed based on diversity to build the ensemble predictor using an iterative approach. In our dynamic ranking and selecting method, a base predictor is initially selected at random. Then in each iteration, all the unselected base predictors are first ranked based on their diversity with the selected base predictor(s), followed by the selection step in which the one with the largest diversity will be added into the set of selected predictors. Diversity between two base classifiers is measured by the proportion of the number of samples with different labels from the two classifiers to the total number of samples in validation dataset. The iteration is terminated when the addition of diversity for the set of selected predictors is less than a specified criterion. The exact stopping criterion for a dataset is determined by a validation dataset which is separated from the dataset of interest. Finally, the selected base predictors are combined to construct an ensemble predictor using a simple majority vote strategy.

## Results and discussion

The purpose of the evaluation is to examine the effectiveness of our proposed PSSM-RT over other methods. Four sets of evaluations are conducted here. Experiment 1 compares PSSM-RT with previous encoding methods. Experiment 2 compares the ensemble learning model with the base classifiers. Experiment 3 compares our proposed predictor EL_PSSM-RT with previous predictors, and Experiment 4 evaluates EL_PSSM-RT on two independent datasets. Based on the obtained data, we further analyse the relation-pairs of amino acids followed a case study of two proteins in the binding-residues identified by our method. In order to assess the significance of statistic comparison between pairs of methods, we calculate the *p*-values of statistic comparisons by the Wilcoxon signed-rank test. As AUC is the most appropriate metric for performance evaluation, for Experiment 1 and Experiment 2, we use AUC to assessed the significance. Because in Experiment 3 and Experiment 4, the AUC values for some methods are not available, so we use ST to calculate the significance. Note that as the evaluation on TS-72 in Experiment 4 have AUC values for models, we use AUC to assessed the significance.

### Window size of PSSM-RT

**Fig. 2:**
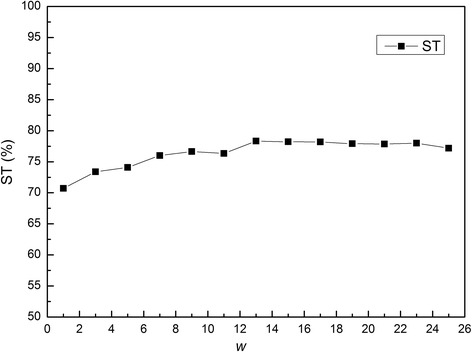
The compact of window size *w* on performance of PSSM-RT. The x-axis is the window size *w* and y-axis is the ST value of PSSM-RT

Since PSSM-RT uses a window based approach, the window size needs to be set properly. For the SVM classifier which uses PSSM-RT as features, the performance of the SVM classifier with different window size *w* is shown in Fig. 2. It can be seen that the ST value continues to increase and peaks when *w* reaches 13. So, the window size *w =* 13 is used for all the SVM classifiers.

### Experiment 1: Comparison of PSSM-RT with previous encoding methods

**Fig. 3:**
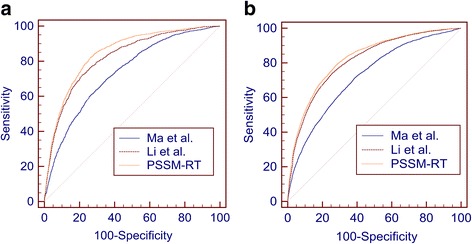
Comparison between different encoding methods. **a** The ROC curves of PSSM-RT, Ma et al.’s method and Li et al.’s method on PDNA-62. **b** The ROC curves of PSSM-RT, Ma et al.’s method and Li et al.’s method on PDNA-224

This set of experiments first compares PSSM-RT to PSSM by using SVM and Random Forest (RF). PSSM-RT is then compared with two types of existing encoding methods: the combination methods and the concatenation methods. The comparison between PSSM-RT and PSSM by using SVM and RF is shown in Table 2. The top performers of the three major performance indicators ST, MCC and AUC are shaded for easy observation. This table shows that PSSM-RT outperforms PSSM significantly with *p*-value of at least less than 2.33e-6 for both SVM and RF on PDNA-62. It also shows that PSSM-RT outperform PSSM significantly on PDNA-224 with *p*-value less than 7.69e-5 for both SVM and RF. As there are a number of combination methods and concatenation methods, we only consider the state-of-the-art works for the respective groups. Consequently, Ma et al.’s work using combination method [56] and Li et al.’s work [32] using the concatenation methods are used for comparison. In Ma et al.’s work, it used PSSM with four physicochemical properties including the lone electron pairs, hydrophobicity, side chain pKa value and molecular mass are combined to calculate the feature representation for residues. In Li et al.’s work, the PSSM scores of residues within the sliding window of the target residue are concatenated to construct the feature vector. So PSSM-RT, Ma et al.’s work and Li et al.’s work all use the same set of features except that Ma et al.’s work uses additional physicochemical features. Both Ma et al.’s work [56] and Li et al.’s work [32] used SVM as the classifier, so we also use SVM as the classifier in this experiment. Note that all the SVM classifiers in this paper used the radial kernel and the parameters of all the SVM classifiers were tuned by the grid method. Since both Ma et al.’s work and Li et al.’s work did not provide the performance for evolutionary features and combination with sequence features on PDNA-62 and PDNA-224, their methods are implemented in this study to obtain evaluation data. The performances on both datasets PDNA-62 and PDNA-224 are shown in Table 3. The corresponding ROC curves are shown in Fig. 3(a) and (b), respectively.

**Table 2:**
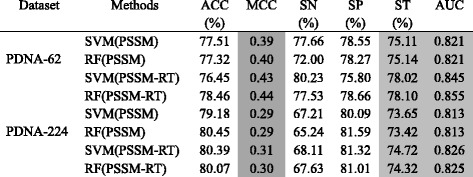
Performance comparison between PSSM-RT and PSSM by multiple classification algorithms on benchmark datasets

**Table 3:** Performance for evolutionary features on benchmark datasets by SVM

Dataset	Methods	ACC (%)	MCC	SN (%)	SP (%)	ST (%)	AUC
PDNA-62	Ma et al.	72.23	0.26	59.45	74.48	66.96	0.734
	Li et al.	77.32	0.40	72.00	78.27	75.14	0.821
	PSSM-RT	76.45	0.43	80.23	75.80	78.02	0.845
PDNA-224	Ma et al.	76.88	0.18	50.59	78.87	64.73	0.723
	Li et al.	79.18	0.29	67.21	80.09	73.65	0.813
	PSSM-RT	80.39	0.31	68.11	81.32	74.72	0.826

From Table 3, we can see that PSSM-RT performs better than Ma et al.’s work on both datasets with *p*-values less than 3.05e-5, which means the improvement is quite significant. More specifically, the increase in the PDNA-62 dataset is 0.17 on MCC, 11.06% on ST and 0.111 on AUC and 0.13 on MCC, 9.99% on ST and 0.103 on AUC for the PDNA-224 dataset. PSSM-RT outperforms Li et al.’s work quite significantly on both datasets with *p*-value less than 4.71e-5. More specifically, the increase in the PDNA-62 dataset is 0.03 on MCC, 2.88% on ST and 0.024 on AUC and 0.02 on MCC, 1.07% on ST and 0.013 on AUC on the PDNA-224 dataset. Figure 3(a) and (b) show that PSSM-RT has the best ROC curve on both PDNA-62 and PDNA-224.

**Fig. 4:**
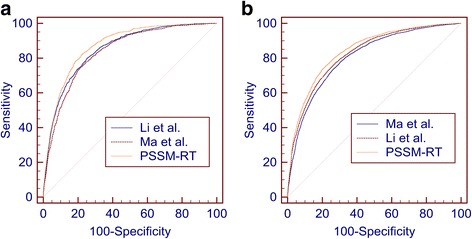
Comparison between different encoding methods when combining with sequence features and physicochemical features. **a** The ROC curves of PSSM-RT, Ma et al.’s method and Li et al.’s method on PDNA-62. **b** The ROC curves of PSSM-RT, Ma et al.’s method and Li et al.’s method on PDNA-224

When both sequence features and physiochemical features are added, the performances of the three methods on PDNA-62 and PDNA-224 are shown in Table 4. The corresponding ROC curves are shown in Fig. 4(a) and (b).

**Table 4:** Performance for all features on benchmark datasets by SVM

Dataset	Methods	ACC (%)	MCC	SN (%)	SP (%)	ST (%)	AUC
PDNA-62	Ma et al.	75.11	0.40	78.22	74.58	76.40	0.837
	Li et al.	77.81	0.42	75.50	78.24	76.87	0.851
	PSSM-RT	81.50	0.48	76.74	82.34	79.54	0.873
PDNA-224	Ma et al.	76.66	0.27	68.95	77.25	73.10	0.808
	Li et al.	78.65	0.29	69.48	79.34	74.41	0.825
	PSSM-RT	78.14	0.31	74.92	78.38	76.65	0.843

Table 4 shows the same performance trends as that in Table 3. Figure 4(a) and (b) also show that PSSM-RT has the best ROC curve on both PDNA-62 and PDNA-224 when the three types of features are combined. This clearly indicates that PSSM-RT outperforms both Ma et al.’s work and Li et al.’s work when all three types of features are used. When comparing Tables 3 and 4, we observe that the performance of PSSM-RT is improved by 0.05 on MCC, 1.52% on ST and 0.028 on AUC for PDNA-62 and 1.92% on ST and 0.017 on AUC for PDNA-224. This shows that PSSM-RT is complementary to the other two features. This set of experiments indicates that the relationships of evolutionary information between residues perform better than the two previous state-of-the-art encoding methods.

### Experiment 2: Comparison of EL_PSSM-RT with base classifiers

**Fig. 5:**
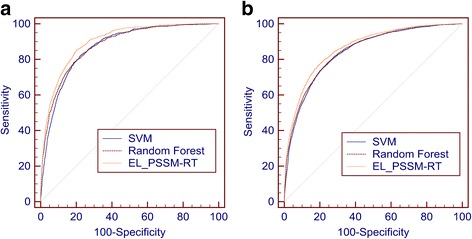
Comparison between EL_PSSM-RT, SVM classifier and Random Forest classifier. **a** The ROC curves EL_PSSM-RT, SVM classifier and Random Forest classifier on PDNA-62. **b** The ROC curves EL_PSSM-RT, SVM classifier and Random Forest classifier on PDNA-224

This set of experiments compares EL_PSSM-RT with the base classifiers. The performances of EL_PSSM-RT, the SVM classifier and the Random Forest (RF) classifier are shown in Table 5, where the performances for the SVM classifier and the RF classifier are their best performances, respectively. Note that EL_PSSM-RT, the SVM classifier and the RF classifier shown in Table 5 are trained from the same set of features. The corresponding ROC curves are shown in Fig. 5(a) and (b).

**Table 5:** Comparison of EL_PSSM-RT with base classifiers on benchmark datasets

Dataset	Methods	ACC (%)	MCC	SN (%)	SP (%)	ST (%)	AUC
PDNA-62	SVM	81.50	0.48	76.74	82.34	79.54	0.873
	RF	80.90	0.47	77.43	81.42	79.43	0.880
	EL_PSSM-RT	80.82	0.51	85.04	80.10	82.57	0.901
PDNA-224	SVM	78.14	0.31	74.92	78.38	76.65	0.843
	RF	80.95	0.32	71.11	81.69	76.40	0.844
	EL_PSSM-RT	78.09	0.34	79.58	77.98	78.78	0.865

Table 5 shows that compares to both the SVM classifier and the RF classifier, EL_PSSM-RT achieves significant performance improvement on both PDNA-62 with *p*-value less than 6.52e-5 and PDNA-224 with *p*-value less than 7.25e-5. More specifically, on the PDNA-62 dataset, the increase to the SVM classifier is 0.03 on MCC, 3.03% on ST and 0.028 on AUC and 0.04 on ACC, 3.14% on ST and 0.021 on AUC to the RF classifier. For the PDNA-224 dataset, the increase to the SVM classifier is 0.03 on MCC, 2.13% on ST and 0.022 on AUC and to the RF classifier is 0.02 on MCC, 2.38% on ST and 0.021 on AUC. Figure 5(a) and (b) also show that EL_PSSM-RT obtains the best ROC curve on both PDNA-62 and PDNA-224. This indicates that ensemble learning makes EL_PSSM-RT more superior than both the SVM classifier and the RF classifier. Furthermore, Table 5 shows that the performance of the RF classifier is good. As RF can examine the learning model and quantify the importance of features used to train the classifier, it helps us to analyze the importance of different features. In this paper, among the 200 top features with the largest importance, we counted the number of features with respect to different categories. The analysis shows that 12 features come from PSSM scores, 134 features come from pair-relationships in PSSM-RT and 26 feature come from multi-relationships in PSSM-RT and 30 come from other features including sequence features and physiochemical features. It validates the importance of pair-relationships and multi-relationships in PSSM-RT for prediction of DNA-binding residues.

For proteins which share less than 25% identical residues, they can may still belong to the same homologous family and have very similar 3D structures, referred to as homologous proteins. Thus, predictors trained on datasets compiled using only the 25% identity threshold can be over-fitted towards large over-represented homologous families. In order to evaluate the influence of homologous proteins on predictors, we construct two novel benchmark datasets based on PDNA-62 and PDNA-224 by keeping only one protein for every homologous families. The novel PDNA-62 contains 35 sequences and the novel PDNA-224 contains 163 sequences. This means that the original PDNA-62 contains approximate 50% homologous proteins and PDNA-224 contains 25% homologous proteins. The results of EL_PSSM-RT, the SVM classifier and the RF classifier on the novel benchmark datasets are shown in Table 6.

**Table 6:** Comparison of EL_PSSM-RT with base classifiers on novel benchmark datasets

Dataset	Methods	ACC (%)	MCC	SN (%)	SP (%)	ST (%)	AUC
PDNA-62	SVM	76.81	0.37	79.65	76.51	78.08	0.861
	RF	79.44	0.36	71.83	80.29	76.06	0.847
	EL_PSSM-RT	80.11	0.43	81.91	79.92	80.92	0.881
PDNA-224	SVM	73.66	0.28	79.68	73.25	76.47	0.839
	RF	76.13	0.27	73.91	76.28	75.10	0.831
	EL_PSSM-RT	76.74	0.32	81.45	76.42	78.93	0.863

Compared to the results on the original benchmark datasets shown in Table 5, the performances of all classifiers are decreased by at least 1.46% ST and 0.012 AUC on novel PDNA-62. On novel PDNA-224, ST and AUC of EL_PSSM-RT and the SVM classifier do not have obvious decrease. But, MCC decrease by at least 0.02. For the RF classifier, ST, AUC and MCC are decreased by 0.05, 1.30% and 0.013, respectively. It indicates that over-representation of some homologous families indeed bias the performance towards those families which leads to biased evaluation. By comparing the performance decreases on PDNA-62 with 50% structure redundant proteins and that on PDNA-224 with 25% redundant proteins, we found that a higher rate of structure redundant proteins leads to more biased evaluation. However, in order to make a fair comparison with state of art methods, we still use the original PDNA-62 and PDNA-224 as datasets for evaluation in the following text.

### Experiment 3: Comparison with previous predictors

This set of experiments evaluates the performance of our proposed ensemble learning based EL-PSSM-RT compared to other state-of-the art methods trained and tested either on PDNA-62 or PDNA-224 including eight algorithms: (1) Dps-pred [14], (2) Dbs-pssm [20], (3) BindN [18], (4) Dp-bind [23], (5) DP-Bind [57], (6) BindN-RF [55], (7) BindN+ [19], and (8) PreDNA [32]. The first seven methods were trained and tested on PDNA-62. The last method, PreDNA [32], was trained and tested on both datasets. PreDNA was proposed recently and achieved the best performance for DNA-binding residue prediction so far. In addition to sequence features and evolutionary information, PreDNA [32] also used structure features. As we have pointed out earlier, structure features of most proteins are unavailable and the experimental 3D structure is very expensive to obtain. Thus, PreDNA [32] cannot be used as a general method at the current time for DNA-binding residue prediction on a genomic scale. For this reason, EL_PSSM-RT does not use any structure feature, similar to many other methods. In order to fairly compare the prediction performance of various methods, the version of PreDNA without using structure features is used in this evaluation. The prediction accuracy of EL_PSSM-RT and other methods on PDNA-62 and PDNA-224 are shown in Tables 7 and 8, respectively.

**Table 7:** Performance comparison of various prediction methods on PDNA-62 by five-fold cross-validation

Methods	ACC (%)	MCC	SN (%)	SP (%)	ST (%)	AUC
Dps-pred	79.10	−	40.30	81.80	61.10	−
Dbs-pssm	66.40	−	68.20	66.00	67.10	−
BindN	70.30	−	69.40	70.50	69.95	0.752
Dp-bind	78.10	0.49	79.20	77.20	78.20	−
DP-Bind	77.20	−	76.40	76.60	76.50	−
BindN-RF	78.20	−	78.10	78.20	78.15	0.861
BindN+	79.00	0.44	77.30	79.30	78.30	0.859
PreDNA^a^	79.40	0.42	76.80	79.70	78.30	−
EL_PSSM-RT	80.82	0.51	85.04	80.10	82.57	0.901

^a^denotes PreDNA without using structure features

**Table 8:** Performance of EL_PSSM-RT Compared with PreDNA on PDNA-224 by five-fold cross-validation

Methods	ACC (%)	MCC	SN (%)	SP (%)	ST (%)	AUC
PreDNA^a^	79.10	0.29	69.50	79.80	74.60	--
EL_PSSM-RT	78.09	0.34	79.58	77.98	78.78	0.865

^a^denotes PreDNA without using structure features

Table 7 shows that EL_PSSM-RT achieves the best performance with significant improvement with *p*-value less than 3.06e-5 for PDNA-62, outperforming other methods by 0.02–0.07 on MCC, 4.27%–21.47% on ST and 0.040–0.149 on AUC. Table 8 shows that, for the PDNA-224 dataset, EL_PSSM-RT performs better than PreDNA by 0.05 on MCC, 4.18% on ST with *p*-value less than 3.64e-5. The results on both datasets indicate that the effect use of relation information and ensemble learning is superior to other existing methods.

### Experiment 4: Independent tests use TS-72 and TS-61

We evaluate the performance of our EL-PSSM-RT on the TS-72 dataset so we can compare it with the previous published DNABR method [33] and the BindN series [18, 19, 55]. DNABR is a sequence based DNA-binding residue prediction method proposed by Ma et al. [33]. BindN, BindN-RF and BindN+ are three methods proposed by Wang et al. using only sequence information [18, 19, 55]. the AUC values of the four published methods are 0.866, 0.748, 0.825 and 0.844, respectively according to Ma et al.’ work [33] which are trained on TR265. The AUC value for EL_PSSM-RT, is 0.879. Our method increases the performance by 0.013–0.131 on AUC with *p*-value less than 8.37e-4 for the independent dataset TS-72.

For the second independent dataset TS-61, we compare our proposed method with DP-Bind[57]. DP-Bind [57] is a web server for predicting DNA-binding sites in a DNA-binding protein from its amino acid sequence. The web server implements three individual machine learning classifiers: DP-Bind(SVM) that uses support vector machine, DP-Bind(KLR) that use kernel logistic regression and DP-Bind(PLR) that uses penalized logistic regression. DP-Bind [57] also implements two types of consensus classifiers. One is majority consensus on the results of three machine learning methods by majority vote, referred to as DP-Bind(MAJ). The other is strict consensus obtained by unanimous agreement, referred to as DP-Bind(STR). The performance of EL_PSSM-RT trained by PDNA-62 and the different DP-Bind methods is shown in Table 9. Table 9 shows that our method outperforms DP-Bind(SVM), DP-Bind(KLR), DP-Bind(PLR) and DP-Bind(MAJ) with 0.05–0.06 on MCC, 3.78–4.43% on ST and 0.027–0.049 on AUC. By comparing to DP-Bind(STR), we found that our method only outperform it marginally. Note that DP-Bind(STR) is based on the assumption that if DP-Bind(SVM), DP-Bind(KLR) and DP-Bind(PLR) assign the same label to a given residue. So it can provide more correct prediction results for residues. The results shown in Table 8 also demonstrates that DP-Bind(STR) achieves better performance than other four models.

**Table 9:** Performance of EL_PSSM-RT Compared with DP-Bind on TS-61

Methods	ACC (%)	MCC	SN (%)	SP (%)	ST (%)	AUC
DP-Bind(SVM)	75.90	0.26	65.99	76.70	71.34	0.794
DP-Bind(KLR)	76.45	0.25	64.22	77.45	70.83	0.790
DP-Bind(PLR)	75.46	0.25	65.24	76.29	70.76	0.812
DP-Bind(MAJ)	76.64	0.26	65.24	77.57	71.41	--
DP-Bind(STR)	80.21	0.31	68.74	81.11	74.92	--
EL_PSSM-RT	77.33	0.31	72.64	77.73	75.19	0.839

However, as DP-Bind(STR) only identify a subset of residues with similar label from the three individual classifiers, it cannot provide prediction results for the other residues. In TS-61, among the 14,253 residues (including 1078 binding residues and 13,175 non-binding residues), DP-Bind(STR) cannot provide prediction results for 2206 residues (including 213 binding residues and 1993 non-binding residues). Therefore, our method is a more general prediction classifier when comparing to DP-Bind(STR).

### Analysis of important pair-relationships

**Fig. 6:**
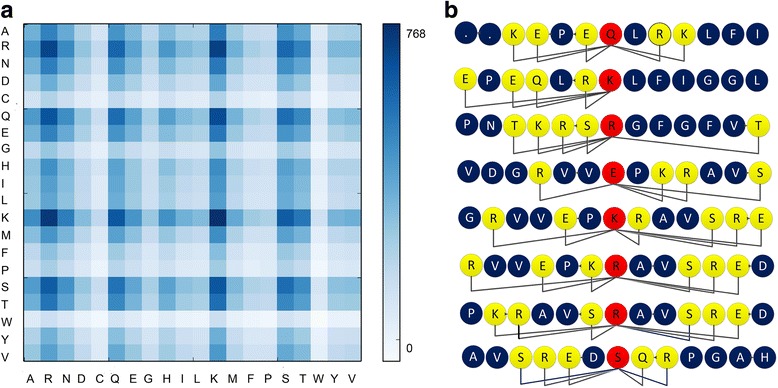
The feature analysis results of PSSM-RT on PDNA-62. **a** The discriminant weights of the 400 features extracted from PSSM-RT. The x axis and y axis denote the 20 residue types. Every element denotes a specific pair-relationship. **b** 6 DNA-binding residues and its context residues extracted from the protein in 1u1q. The red residues are the binding residues and the yellow ones are the residues that can form important pair-relationship with it. The rest ones are the unimportant residues. The black polyline are the important pair-relationships

To further understand the importance of PSSM-RT for DNA-binding residue prediction, we analyze the important pair-relationships found by the learning algorithm. Since the importance of the relations can be reflected by the discriminant weight vector of the pair-relationships extracted by PSSM-RT, the values in the discriminant weight vector indicates the discriminant powers of the features in the feature space. Following the published works in [58–60], the discriminant weight vector **W** is calculated as follows: first, we obtain the classification weight vector **A** from the ensemble learning classifier during the training process. **W** is calculated by applying the following formulae:

14 $$ \mathbf{W}={\mathbf{A}}^{\mathrm{T}}\cdot \mathbf{M}={\left[\begin{array}{c}\hfill {a}_1\hfill \\ {}\hfill {a}_2\hfill \\ {}\hfill \vdots \hfill \\ {}\hfill {a}_n\hfill \end{array}\right]}^{\mathrm{T}}\left[\begin{array}{cccc}\hfill {m}_{11}\hfill & \hfill {m}_{12}\hfill & \hfill \cdots \hfill & \hfill {m}_{1d}\hfill \\ {}\hfill {m}_{21}\hfill & \hfill {m}_{22}\hfill & \hfill \cdots \hfill & \hfill {m}_{2d}\hfill \\ {}\hfill \vdots \hfill & \hfill \vdots \hfill & \hfill \cdots \hfill & \hfill \vdots \hfill \\ {}\hfill {m}_{n1}\hfill & \hfill {m}_{n2}\hfill & \hfill \cdots \hfill & \hfill {m}_{nd}\hfill \end{array}\right] $$

where **A** is the classification weight vector of the training dataset by the ensemble learning classifier trained on PDNA-62 and **M** is the feature vectors of all training data instances; *d* is the dimension of the feature space and *n* is the number of data instances in the training dataset. The analysis results are shown in Fig. 6 based on the data given in the part E of the Additional file 1 which lists all the discriminant weights of the 400 pair-relationships between the target residue and its neighboring residue. Figure 6 includes a heatmap showing the discriminant weight of every pair-relationship and a diagram of binding residues showing the pair-relationships between important residues. Figure 6a shows that the relationships between amino acid pairs (K, K), (K, R), (R, R), (Q, K), (Q, R), (S, K), (S, R), (R, Q), (S, S), (S, Q), (T, R), (E, K), (E, R), (E, R),(E, Q) are the fifteen relationships with larger discriminant weights. This means that the amino acids K, R, Q, S, T and E are important in the interaction between proteins and its corresponding DNA molecular. This feature analysis result is consistent with many other works for DNA-binding proteins research which stated that R, K, E and S are important for the interaction between DNA-binding proteins and its responsible DNA molecules [61, 62]. Figure 6b shows eight DNA-binding residues and its context residues extracted from the structure of a protein-DNA complex (PDB id: 1u1q). As we can see from Fig. 6b, the relationship between R and K has the highest occurrence frequency among the eight DNA-binding residues and is the most important feature for DNA-binding residue prediction for this protein. The second most important feature is the relationship between R and K. The relationships between E and Q and between E and R are the third most important features. The analysis result validates the usefulness of PSSM-RT for the representation of DNA-binding residues.

### Case study

**Fig. 7:**
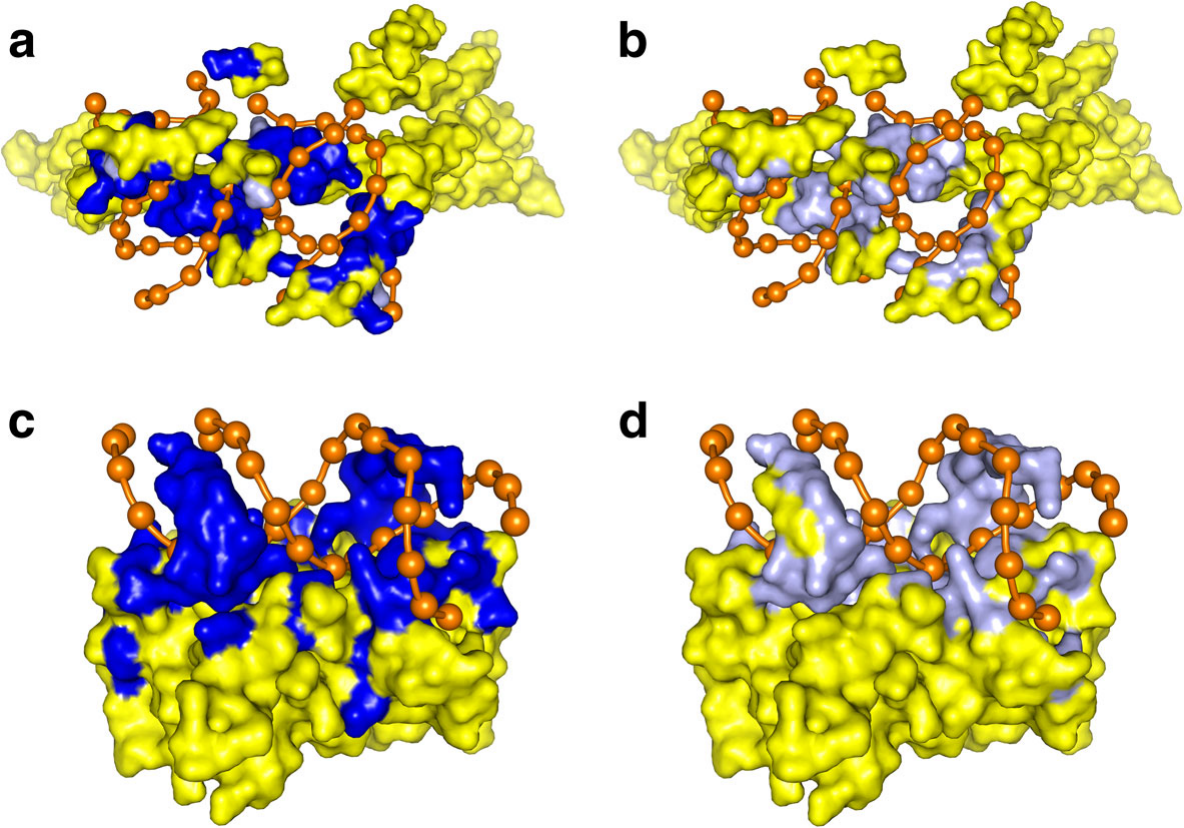
Actual residues and predicted residues of proteins in 1s40 and 1b3t. **a** The predicted binding residues on the protein in 1s40. **b** The actual binding residues on the protein in 1s40. **c** The predicted binding residues on the protein in 1b3t. **d** The actual binding residues on the protein in 1b3t

In order to further validate the usefulness of EL_PSSM-RT for DNA-binding residue prediction, we apply EL_PSSM-RT trained on PDNA-62 to distinguish the binding residues from non-binding residues for two protein-DNA complexes which are not in the training set, namely, 1s40 and 1b3t. The proteins in these two complexes are two typical DNA-binding proteins and the sequences have sequence similarity of less than 25% for all the sequences in the training set. On 1s40, EL_PSSM-RT achieves 96.71% on ACC, 0.74 on MCC, 92.06% on SN, 96.96% on SP and 94.51% on ST, respectively. This means that 34 residues out of a total of 39 actual binding residues are correctly predicted by EL_PSSM-RT and only 24 residues in the 264 non-binding residues are incorrectly predicted as binding residues. The actual residues and predicted residues in 1s40 are shown in Fig. 7a and b, respectively. The two figures show that most of the real binding residues overlap with the predicted binding residues. This provides a visual indication that most of binding residues are correctly predicted.. In the case of 1b3t, EL_PSSM-RT achieves 90.02% on ACC, 0.60 on MCC, 79.17% on SN, 91.35% and 85.25% on ST, respectively. In other words, 40 residues out of 48 binding residues are correctly predicted and only 32 residues out of 244 non-binding residues are incorrectly predicted as binding residues. Figure 7c and d depict the actual binding regions and predicted binding regions on 1b3t, respectively. We can see that most of the actual binding residues overlap with the predicted binding residues and only very few non-binding residues are wrongly identified as the binding residues.

### Web service description

A user-friendly web service of EL_PSSM-RT is made available in order to make our proposed predictor freely accessible to the public. For the convenience of users, we provide a step-by-step guideline to use EL_PSSM-RT below.

**Fig. 8:**
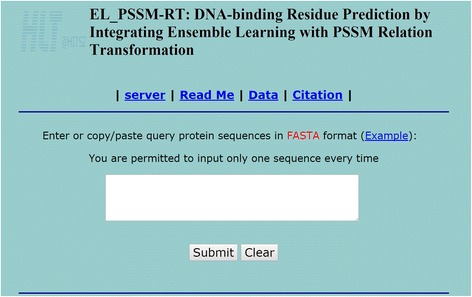
The homepage of the web service of EL_PSSM-RT. The web address of this webserver is http://hlt.hitsz.edu.cn:8080/PSSM-RT_SVM/. See the description in the server description for further explanation

- **Step 1.** Using the URL (http://hlt.hitsz.edu.cn:8080/PSSM-RT_SVM/) to access the web service (as shown in Fig. 8). The **Read Me** button on the homepage supplies more details of EL_PSSM-RT.
- **Step 2**. Type or copy and paste a query sequence in the input box at the center of the homepage. The query sequence must be in the fasta format [63]. By clicking the **Example button**, some examples for sequences in the fasta format [63] will be returned. As EL_PSSM-RT needs to apply PSI_BLAST, PSIPRED and SABLE to retrieve PSSMs, predicted secondary structures as well as predicted solvent accessible area for the query sequence, it will take quite some time. Therefore, only one sequence is allowed for submission at a time.
- **Step 3.** Click the **Submit** button to get the prediction from the server. The predicted results of all residues in the sequence will be displayed on the result page, where ‘+’ denotes a binding residue and ‘-’ denotes a non-binding residue.

## Conclusion

As the interactions between proteins and DNAs are mainly formed by the immediate contacts between them, the identification of residues involved in the contacts is important for understanding the mechanism between amino acids and nucleotides. Many methods have been proposed to use evolutionary information for DNA-binding residues prediction. The combination methods and the concatenation methods are two commonly used methods. Both of them used only the evolutionary information of residues, yet the relationships of evolutionary information between residues are overlooked. In this paper, we propose a novel PSSM encoding method, referred to as PSSM-RT, which includes the relationships of evolutionary information between residues. On both the PDNA-62 dataset and the PDNA-224 dataset, PSSM-RT performs better than the combination methods and the concatenation methods. When sequence features and physicochemical features are added, the prediction performance is further improved. This indicates that the evolution information, sequence features and physiochemical features are complementary for predictions. By combining ensemble learning and PSSM-RT, we propose a novel classifier EL_PSSM-RT to better handle the imbalance between binding and non-binding residues in datasets. The comparison of EL_PSSM-RT with the SVM classifier and the RF classifier on PDNA-62 and PDNA-224 indicates that ensemble learning is indeed useful for DNA-binding residue prediction. Performance comparisons between EL_PSSM-RT and existing predictors on two commonly used datasets and two independent datasets demonstrate that EL_PSSM-RT is more effective than the published works. Feature analysis of PSSM-RT on the PDNA-62 dataset demonstrates that PSSM-RT can extract many useful pair-relationships for DNA-binding residue prediction. The case study on 1s40 and 1b3t indicates that EL_PSSM-RT can correctly predict most of the binding residues with very low false positive rate. The performance evaluation and the case study on 1s40 and 1b3t show that the relationship of evolutionary information between residues is indeed useful in DNA-binding residue prediction and ensemble learning can be used to address the data imbalance issue between binding and non-binding residues in training datasets. Furthermore, we construct two novel benchmark datasets based on PDNA-62 and PDNA-224 by keeping only one protein for every homologous families and evaluate EL_PSSM-RT as well as the SVM classifier and the RF classifier on these two novel benchmark datasets. By comparing the performance on the novel benchmark datasets with that on the original benchmark datasets, we observe that over-representation of some homologous families indeed bias the performance towards those families. So our future works will study the details of DNA-binding residues for homologous proteins and the influence of homologous proteins on the predicting performance of EL_PSSM-RT and state-of-the-art methods.

## Additional file

Additional file 1: The PDB id and the chain id of the protein sequences in the four datasets and the discriminant weights of the 400 pair-relationships between the target residue and its neighboring residue extracted from PSSM-RT. (DOC 141 kb)

## Abbreviations

ACC: Accuracy
AUC: Area under ROC curve
ChIP: Chromatin immunoprecipitation
EIIP: Electron-ion interaction potential
EMSAs: Electrophoretic mobility shift assays
KLR: Kernel logistic regression
LEPs: Lone electron pairs
MAJ: Majority vote
MCC: Mathews Correlation Coefficient
NMR: Nuclear magnetic resonance
NR: Non-redundant
PAIR: Peptide nucleic acid-assisted identification of RNA binding proteins
PDB: Protein Data Bank
PLR: Penalized logistic regression
PNA: Peptide nucleic acid
PSSM: Position Specific Score Matrix
PSSM-RT: Position Specific Score Matrix Relation Transformation
RBPs: RNA binding proteins
RF: Random Forest
ROC: Receiver Operating Characteristic
SN: Sensitivity
SP: Specificity
ST: Strength
STR: Strict consensus
SVM: Support Vector Machine
