# Supplementary material for: EL_PSSM-RT: DNA-binding residue prediction by integrating ensemble learning with PSSM Relation Transformation
Source: BMC Bioinformatics. 2017 Aug 29;18:379. doi: 10.1186/s12859-017-1792-8 (PMC5576297; doi:10.1186/s12859-017-1792-8)
Supplement: Additional file 1: — The PDB id and the chain id of the protein sequences in the four datasets and the discriminant weights of the 400 pair-relationships between the target residue and its neighboring residue extracted from PSSM-RT. (DOC 141 kb) [file 12859_2017_1792_MOESM1_ESM.doc]

# EL_PSSM-RT: DNA-binding Residue Prediction by Integrating Ensemble Learning with PSSM Relation Transformation

Jiyun Zhou^1, 2^, Qin Lu^2^, Ruifeng Xu^1, 3 *^, Yulan He^4^, Hongpeng Wang^1^

1 School of Computer Science and Technology, Harbin Institute of Technology Shenzhen Graduate School, Shenzhen, Guangdong, China

2 Department of Computing, the Hong Kong Polytechnic University, Hong Kong

3 Shenzhen Engineering Laboratory of Performance Robots at Digital Stage, Shenzhen Graduate School, Harbin Institute of Technology, Shenzhen, China

4 School of Engineering and Applied Science, Aston University, United Kingdom

* Corresponding authors

**Part A** The PDB id and the chain id of the protein sequences in PDNA-62.

>1A02:N >1A02:F >1A02:J >1BL0:A

>1DP7:P >1HDD:C >1MDY:A >1PER:L

>1TC3:C >1A74:A >1C0W:B >1ECR:A

>1HLO:A >1MEY:F >1PNR:A >1TF3:A

>1AAY:A >1CDW:A >1GAT:A >1HRY:A

>1MHD:A >1PUE:E >1TRO:A >1AZQ:A

>1CF7:A >1FJL:A >1HWT:D >1MNM:A

>1MNM:C >1PVI:B >1TSR:A >1B3T:A

>1CJG:A >1GCC:A >1IF1:A >1MSE:C

>1PYI:A >1UBD:C >1J59:A >1CMA:A

>1GDT:A >1IGN:A >1OCT:C >1REP:C

>1XBR:A >1BF5:A >1D02:A >1HCQ:A

>1IHF:A >1IHF:B >1PAR:B >1SRS:A

>1YRN:A >1YRN:B >1BHM:A >1D66:A

>1HCR:A >1LMB:4 >1PDN:C >1SVC:P

>1YSA:C >1YUI:A >2BOP:A >2DRP:D

>2HDC:A >2GLI:A >3CRO:L

**Part B** The PDB id and the chain id of the protein sequences in PDNA-224.

>1A02:N >1A02:F >1A35:A >1A73:A

>1B01:A >1B3T:A >1B72:B >1BDH:A

>1BG1:A >1BPX:A >1BRN:L >1C9B:A

>1CBV:L >1CMA:A >1CW0:A >1D02:A

>1D2I:A >1D5Y:A >1DC1:A >1DDN:A

>1DH3:A >1DMU:A >1DNK:A >1DP7:P

>1EBM:A >1ECR:A >1EMH:A >1EOO:A

>1ESG:A >1EYU:A >1F4K:A >1F4R:A

>1FIU:A >1FJX:A >1FYM:B >1FZP:B

>1GD2:E >1GDT:A >1H38:A >1H8A:C

>1HCR:A >1HHT:P >1HJB:A >1HJB:C

>1HLV:A >1HWT:C >1I3J:A >1I7D:A

>1IAW:A >1IGN:A >1J1V:A >1JB7:A

>1JE8:A >1JEY:B >1JEY:A >1JMC:A

>1JNM:A >1K78:A >1KC6:A >1L3L:A

>1LLI:A >1LQ1:A >1MA7:A >1MDY:A

>1MM8:A >1N6J:A >1NKP:A >1NOP:A

>1ODH:A >1OUP:A >1OUZ:A >1P4E:A

>1P7D:A >1PAR:A >1Q9Y:A >1R71:A

>1R7M:A >1RBJ:A >1REP:C >1RH6:A

>1RRQ:A >1SA3:A >1SFU:A >1T2K:D

>1T8E:B >1TAU:A >1TGH:A >1TRO:A

>1TSR:A >1U1K:A >1U3E:M >1U78:A

>1U8B:A >1UUT:A >1VAS:A >1WVL:A

>1X9N:A >1XC8:A >1XF2:H >1XPX:A

>1XSD:A >1YFJ:A >1Z63:A >1ZNS:A

>1ZX4:A >1ZZI:A >2A0I:A >2A3V:A

>2BOP:A >2BPA:1 >2C5R:B >2C62:A

>2C9L:Y >2CGP:A >2D7D:A >2DGC:A

>2ES2:A >2ETW:A >2EX5:A >2EZV:A

>2F8N:K >2FIO:A >2FKC:A >2FQZ:A

>2H1O:E >2H1O:A >2H27:A >2H7F:X

>2HAN:B >2HVS:A >2I0Q:B >2I13:A

>2NNY:A >2NTC:A >2O49:A >2O61:A

>2O8B:B >2OWO:A >2OXV:A >2P6R:A

>2PY5:A >2Q2K:A >2Q2T:A >2QFJ:A

>2QHB:A >2QL2:D >2QL2:A >2QSH:A

>2R9L:A >2RBF:A >2RGR:A >2VHG:A

>2VS8:A >2W42:A >2W7N:A >2WTF:A

>2XSD:C >2YVH:A >2Z3X:A >2ZO0:B

>3A5T:A >3A5U:A >3A6N:A >3A6N:B

>3A6N:D >3AAF:A >3B39:A >3BRF:A

>3BS1:A >3C25:A >3C2I:A >3C2P:A

>3CLC:A >3COQ:A >3CRO:L >3D6Y:A

>3DLB:A >3DSC:A >3E6C:C >3EH8:A

>3EI1:B >3ERE:D >3EY1:A >3EYI: A

>3F27:D >3F2B:A >3FD2:A >3FDQ:A

>3FMT:A >3G00:A >3GQC:A >3GX4:X

>3GXQ:A >3GZ6:A >3H0D:A >3H25:A

>3H8R:A >3HQF:A >3I2O:A >3IAY:A

>3IGM:A >3IV5:A >3JSO:A >3K4X:A

>3KDE:C >3KET:A >3KJO:A >3KK1:A

>3KMP:A >3KNT:A >3KYL:A >3L2R:A

>3LWH:A >3M4A:A >3M7K:A >3MAQ:A

>3MKW:B >3MVA:O >3MVD:K >3N1I:A

>3OA6:A >3OD8:A >3ORC:A >3OSN:A

**Part C** The PDB id and the chain id of the protein sequences in TS-72.

>1F0V:A >2OFI:A >1HBX: A >2PJR:B

>1QZG:A >3PJR:A >1FOK:A >1DC1:A

>1Y1W:G >1RZ9:A >1M06:G >1NKP:A

>1FIU:A >1U3E:M >1AKH:A >1AKH:B

>1QN3:A >1D2I:A >2ASD:A >1NGM:B

>1NLW:A >1C9B:A >1RRQ:A >1JNM:A

>1GD2:E >1K61:A >1A6Y:A >1AWC:A

>1AWC:B >2C6Y:A >1CKQ:A >2BSQ:E

>1LAU:E >1HAO:H >1RZR:L >1CIT:A

>2JEA:I >1JT0:A >1IAW:A >1SKN:P

>1TQE:P >1AOI:A >1AOI:B >1AOI:D

>1EO3:A >2H7G:X >2EZV:A >1B2M:A

>2A66:A >2B9S:A >2B9S:B >1HHT:P

>1CW0:A >1FOS:E >2EX5:A >1FZP:B

>1MOW:A >1X9N:A >3E6C:C >1D3U:B

>3N6S:A >1A36:A >1ODH:A >1D8Y:A

>1I6H:C >1I6H:E >1I6H:F >1I6H:H

>1I6H:I >1I6H:J >1I6H:K >1I6H:L

**Part D** The PDB id and the chain id of the protein sequences in TS-61.

>1AKH:A >1IV6:A >1NG9:A >1NK2:P

>1NNE:A >1PH1:B >1QZG:A >1S6M:A

>1Z1B:A >2BNZ:A >2KKF:A >2LTT:A

>2MNA:A >2NP2:A >2O8B:A >2XMA:A

>2YPF:A >3HOS:A >3ODC:A >3ON0:A

>3POV:A >3PVP:A >3QMB:A >3SQI:A

>3SSC:A >3TED:A >3THW:B >3U58:A

>3U5Z:A >3ULP:A >3V20:A >3VDY:A

>3VEA:A >3VKE:A >3VW4:A >4ATK:A

>4DWP:A >4ER8:A >4F6M:A >4FCY:A

>4HID:A >4IX7:A >4JBM:A >4JJN:D

>4KPY:A >4LVI:A >4MTD:A >4NDF:A

>4PAR:A >4R2A:A >4UMK:A >4WCG:A

>4WZW:A >4Y00:A >4Z3C:C >4Z47:A

>5A3D:A >5BPD:A >5EYB:A >5FGP:A

>5ITR:A

**Part E** The discriminant weights of the 400 pair relationships between the target site and its neighboring site extracted from PSSM-RT.

| Pair | KK | KR | RK | RR | QK | QR | SK | KQ | SR | KS |
| --- | --- | --- | --- | --- | --- | --- | --- | --- | --- | --- |
| Weight | 767.9 | 744.9 | 719.9 | 709.4 | 691.7 | 670.2 | 662.8 | 660.0 | 648.9 | 647.8 |
| Pair | RQ | RS | QQ | SS | QS | TK | SQ | TR | KT | EK |
| Weight | 623.8 | 615.0 | 604.6 | 602.0 | 601.7 | 598.9 | 587.5 | 581.9 | 575.5 | 573.4 |
| Pair | NK | AK | NR | KN | ER | RT | AR | TS | QT | TQ |
| Weight | 571.2 | 558.8 | 554.9 | 553.0 | 546.2 | 541.6 | 541.4 | 531.9 | 528.5 | 527.7 |
| Pair | RN | ST | KA | HK | MK | QN | NS | HR | KH | AS |
| Weight | 522.1 | 518.7 | 515.6 | 514.6 | 511.9 | 511.2 | 510.0 | 505.6 | 504.1 | 503.3 |
| Pair | NQ | SN | EQ | ES | AQ | MR | KE | RA | IK | RH |
| Weight | 502.9 | 501.9 | 496.4 | 495.5 | 494.6 | 489.0 | 489.0 | 486.4 | 486.4 | 485.1 |
| Pair | VK | QA | SA | TT | LK | IR | QH | HS | MQ | MS |
| Weight | 480.2 | 476.2 | 468.9 | 462.2 | 461.8 | 459.7 | 458.2 | 455.4 | 455.0 | 453.7 |
| Pair | VR | RE | SH | MW | HQ | TN | NT | SE | LR | ET |
| Weight | 453.5 | 452.0 | 451.0 | 450.4 | 449.5 | 444.8 | 438.8 | 438.1 | 437.8 | 436.4 |
| Pair | IQ | DK | KM | AT | NN | VS | IS | VQ | AN | EN |
| Weight | 432.3 | 430.5 | 429.7 | 429.7 | 429.2 | 428.7 | 428.5 | 425.9 | 422.9 | 422.4 |
| Pair | LS | DR | LQ | TA | KV | YK | TH | KI | AA | RM |
| Weight | 417.6 | 413.1 | 411.4 | 410.1 | 409.2 | 408.7 | 407.8 | 405.1 | 405.1 | 404.1 |
| Pair | HT | YR | EA | NA | QM | EE | MT | KY | TE | MN |
| Weight | 400.4 | 399.6 | 398.9 | 398.1 | 396.8 | 394.8 | 394.7 | 394.2 | 393.3 | 389.9 |
| Pair | DS | NH | RV | HN | RI | NE | SM | RY | AE | QV |
| Weight | 386.9 | 383.2 | 382.7 | 382.4 | 381.5 | 380.1 | 379.4 | 379.3 | 378.2 | 376.7 |
| Pair | IT | DQ | VT | QI | KL | EH | YS | AH | KD | IN |
| Weight | 375.6 | 374.6 | 374.1 | 373.9 | 371.7 | 369.5 | 369.1 | 368.5 | 367.7 | 366.6 |
| Pair | FK | SV | VN | LT | MA | HH | YQ | QY | SY | ME |
| Weight | 363.2 | 362.2 | 362.1 | 362.1 | 361.4 | 360.4 | 359.4 | 357.4 | 354.3 | 353.1 |
| Pair | SI | LN | FR | RL | HA | GK | QL | RD | QD | MH |
| Weight | 353.0 | 353.0 | 350.9 | 349.6 | 347.7 | 344.5 | 343.8 | 343.6 | 339.4 | 337.8 |
| Pair | SD | TM | GR | VA | DT | KG | SL | IE | FS | IA |
| Weight | 337.0 | 336.7 | 336.1 | 335.2 | 333.2 | 332.7 | 332.5 | 332.1 | 332.0 | 332.0 |
| Pair | VE | HE | EM | NM | LA | DN | AM | TV | FQ | IH |
| Weight | 331.3 | 331.3 | 329.2 | 328.6 | 327.7 | 324.9 | 324.3 | 324.0 | 321.6 | 321.6 |
| Pair | LE | KF | VH | YT | TI | EV | TY | GS | PK | YN |
| Weight | 320.6 | 320.3 | 319.3 | 317.4 | 316.3 | 315.1 | 315.0 | 314.9 | 314.7 | 314.4 |
| Pair | NV | RG | DA | EI | NY | LH | AV | RF | NI | QG |
| Weight | 308.9 | 308.2 | 308.0 | 306.6 | 305.6 | 305.6 | 303.7 | 302.7 | 302.7 | 302.4 |
| Pair | SG | PR | GQ | DE | TD | ED | HM | AI | TL | QF |
| Weight | 301.8 | 301.2 | 298.2 | 297.7 | 297.0 | 295.4 | 295.0 | 294.6 | 292.5 | 292.5 |
| Pair | KP | ND | AY | AD | SF | EY | EL | MM | NL | FT |
| Weight | 292.0 | 291.5 | 289.8 | 289.0 | 288.8 | 287.3 | 287.3 | 287.1 | 285.6 | 284.1 |
| Pair | CR | HV | CK | YA | AL | YH | FN | HI | DH | HY |
| Weight | 282.3 | 282.1 | 281.8 | 281.6 | 281.6 | 280.7 | 280.6 | 279.8 | 279.6 | 279.1 |
| Pair | PS | GT | MV | SP | TG | IM | VM | RP | PQ | QP |
| Weight | 278.7 | 272.6 | 272.3 | 270.2 | 269.2 | 268.8 | 268.7 | 267.7 | 267.7 | 267.2 |
| Pair | YE | MD | MI | VV | GN | IV | CS | EG | NG | AG |
| Weight | 267.1 | 266.4 | 264.5 | 260.3 | 259.9 | 258.6 | 258.5 | 258.3 | 258.2 | 257.9 |
| Pair | HL | MY | FA | LM | HD | TF | II | ID | VI | KC |
| Weight | 256.2 | 256.1 | 255.0 | 254.6 | 254.1 | 253.6 | 250.7 | 250.5 | 249.0 | 248.9 |
| Pair | GA | VD | DM | NF | CQ | FE | LV | FH | VY | ML |
| Weight | 247.8 | 247.6 | 247.2 | 246.4 | 246.2 | 246.0 | 245.9 | 245.4 | 244.7 | 244.2 |
| Pair | TP | RC | IY | LD | EF | PT | AF | DV | LI | PN |
| Weight | 243.8 | 243.4 | 243.0 | 242.9 | 242.5 | 242.3 | 241.4 | 236.7 | 236.2 | 234.6 |
| Pair | EP | MG | NP | VL | GE | DI | GH | IL | SC | DD |
| Weight | 234.4 | 232.5 | 232.0 | 230.7 | 230.3 | 229.5 | 229.1 | 229.0 | 228.8 | 227.1 |
| Pair | LY | AP | YM | QC | HG | HF | VG | DY | PA | WK |
| Weight | 226.4 | 226.2 | 226.1 | 225.5 | 225.3 | 223.8 | 223.7 | 222.7 | 220.0 | 219.4 |
| Pair | CT | IG | DL | LL | YV | CN | WR | PE | LG | YI |
| Weight | 219.3 | 217.8 | 217.8 | 217.4 | 217.3 | 216.9 | 213.7 | 213.2 | 213.0 | 211.0 |
| Pair | YY | KW | YD | MF | HP | MP | PH | VF | FM | TC |
| Weight | 210.9 | 208.6 | 207.4 | 207.2 | 204.2 | 204.1 | 204.1 | 200.7 | 199.8 | 199.4 |
| Pair | VP | RW | DG | CH | GM | CA | NC | IF | FV | WS |
| Weight | 199.3 | 199.2 | 198.7 | 198.3 | 197.8 | 196.1 | 195.0 | 194.5 | 193.7 | 193.0 |
| Pair | YL | IP | GV | WQ | FD | DF | LP | QW | AC | YG |
| Weight | 192.7 | 192.4 | 191.4 | 190.9 | 190.1 | 187.2 | 186.4 | 186.3 | 185.8 | 185.0 |
| Pair | DP | FI | FY | SW | EC | HC | GI | GY | LF | CE |
| Weight | 184.9 | 184.2 | 184.2 | 183.4 | 183.3 | 182.2 | 181.8 | 181.7 | 181.6 | 180.5 |
| Pair | GD | GG | PM | GL | PV | YF | FL | FG | WN | YP |
| Weight | 177.7 | 173.7 | 172.2 | 172.0 | 169.1 | 168.8 | 168.2 | 167.7 | 166.2 | 166.0 |
| Pair | PD | WT | MC | NW | TW | PY | CM | PG | CY | PI |
| Weight | 165.1 | 164.4 | 164.1 | 162.2 | 162.1 | 159.1 | 158.4 | 157.5 | 157.3 | 156.6 |
| Pair | IC | EW | AW | VC | GF | FP | PL | WA | GP | CV |
| Weight | 152.5 | 151.8 | 151.7 | 150.9 | 150.7 | 149.9 | 149.2 | 149.1 | 149.0 | 148.4 |
| Pair | LC | FF | WH | DC | YC | CI | PP | HW | CD | WE |
| Weight | 148.4 | 147.7 | 147.3 | 147.2 | 147.1 | 146.4 | 145.6 | 145.3 | 143.8 | 143.6 |
| Pair | CL | MW | PF | FC | CF | CG | VW | IW | GC | WM |
| Weight | 137.9 | 134.2 | 131.2 | 131.1 | 130.3 | 128.8 | 126.2 | 124.7 | 122.9 | 119.0 |
| Pair | DW | LW | CC | WY | YW | WV | CP | WD | WI | PC |
| Weight | 117.3 | 116.9 | 116.2 | 113.8 | 112.3 | 112.2 | 111.0 | 110.1 | 107.9 | 101.8 |
| Pair | FW | WL | WG | GW | WF | CW | WP | WC | PW | WW |
| Weight | 100.9 | 100.1 | 97.8 | 96.3 | 94.0 | 85.9 | 83.3 | 80.5 | 80.3 | 59.8 |
